# First-line atezolizumab/durvalumab plus platinum–etoposide combined with radiotherapy in extensive-stage small-cell lung cancer

**DOI:** 10.1186/s12885-023-10784-8

**Published:** 2023-04-06

**Authors:** Lijuan Li, Dan Yang, Yanmei Min, Anyan Liao, Jing Zhao, Leilei Jiang, Xin Dong, Wei Deng, Huiming Yu, Rong Yu, Jun Zhao, Anhui Shi

**Affiliations:** 1 Key Laboratory of Carcinogenesis and Translational Research (Ministry of Education/Beijing), Department of Radiation Oncology, Peking University Cancer Hospital and Institute, No. 52 Fucheng Road, Haidian District, Beijing, 100142 China; 2 Department of Oncology, The Third Hospital of Mianyang (Sichuan Mental Health center), Mianyang, China; 3 Department of Radiation Oncology, Beijing United Family Medical Center (New Hope), Beijing, China; 4 Key Laboratory of Carcinogenesis and Translational Research (Ministry of Education/Beijing), Department I of Thoracic Oncology, Peking University Cancer Hospital and Institute, Beijing, China

**Keywords:** Extensive-stage small-cell lung cancer, Immunotherapy, Radiotherapy

## Abstract

**Background:**

Immunotherapy has made significant advances in the treatment of extensive-stage small-cell lung cancer (ES-SCLC), but data in combination with radiotherapy are scarce. This study aims to assess the safety and efficacy of chemoimmunotherapy combined with thoracic radiotherapy in patients with ES-SCLC.

**Methods:**

This single-center retrospective study analyzed patients with ES-SCLC who received standard platinum–etoposide chemotherapy combined with atezolizumab or durvalumab immunotherapy as induction treatment, followed by consolidative thoracic radiotherapy (CTRT) before disease progression in the first-line setting. Adverse events during radiotherapy with or without maintenance immunotherapy and survival outcomes were assessed.

**Results:**

Between December 2019 and November 2021, 36 patients with ES-SCLC were identified to have received such treatment modality at one hospital. The number of metastatic sites at diagnosis was 1–4. The biological effective dose of CTRT ranged from 52 to 113 Gy. Only two patients (6%) developed grade 3 toxic effect of thrombocytopenia, but none experienced grade 4 or 5 toxicity. Four patients developed immune-related pneumonitis during the induction treatment period but successfully completed later CTRT. The rate of radiation-related pneumonitis was 8% with grades 1–2 and well tolerated. The median progression-free survival (PFS) was 12.8 months, but the median overall survival (OS) was not determined. The estimated 1-year OS was 80.2% and 1-year PFS was 53.4%.

**Conclusions:**

Immunotherapy combined with CTRT for ES-SCLC is safe and has ample survival benefit.

## Background

Small-cell lung cancer (SCLC) remains a challenging disease, accounting for approximately 15% of all lung cancers [1]. SCLC is characterized by rapid tumor growth, high vascularity, and early metastatic dissemination, making it a highly aggressive systemic malignancy; two-thirds of patients present with extensive-stage SCLC (ES-SCLC) at diagnosis [2, 3] .

ES-SCLC has an extremely poor outcome, and for more than three decades, its standard first-line treatment is platinum–etoposide chemotherapy. Relapse is frequent despite the initial response and median overall survival (OS) of merely 10 months [3]. Recent therapeutic clinical advances in immunotherapy have been reported. For example, both the IMpower133 trial (atezolizumab) [4] and CASPIAN trial (durvalumab) [5] demonstrated that immune checkpoint inhibitors (ICI) in addition to first-line chemotherapy prolongs OS for 2–3 months in untreated ES-SCLC.

Limited-stage SCLC is radiosensitive in nature, so radiotherapy (RT) has always been the mainstay. For ES-SCLC, the use of RT is inconsistent worldwide. Jeremic et al. first found that patients with ES-SCLC receive a survival benefit for the addition of consolidative thoracic radiotherapy (CTRT) compared to those undergoing chemotherapy alone [6]. More recently, the large CREST phase III trial by Slotman et al. showed that CTRT accompanied by prophylactic cranial irradiation significantly improved the 2-year OS and reduced the progression rate of patients with ES-SCLC who respond to chemotherapy [7]. Prospective RTOG 0937 study also showed delayed disease progression but no significant OS improvement [8].

In the era of chemoimmunotherapy, the role of RT for ES-SCLC should be reintroduced because of the encouraging synergistic antitumor effect between radiation therapy and immunotherapy [9]. Growing preclinical and clinical evidence suggest that RT be combined with immunomodulators, in particular ICI such a PD-1/PD-L1 inhibitor [10]. RT can deeply reshape the immune microenvironment by increasing tumor antigen exposure and regulating T-cell infiltration, which when combined with ICI amplify immune response and improve efficacy [9, 11]. Durvalumab after chemoradiotherapy for stage III unresectable non-small cell lung cancer have been proved as safe and effective treatment paradigm [12].

To the best of our knowledge, there are two randomized trials (NCT04402788 [13] and NCT04462276 [14]) addressing the safety and efficacy of RT-immunotherapy combination for patients with ES-SCLC, but no results have been reported yet. CTRT is not designed in IMpower133 and CASPIAN trials, and patients in these trials had treatment-resistant disease. Moreover, immunotherapy only modestly improves survival. CTRT has positive effects in ES-SCLC and the potential synergy of RT-immunotherapy modalities. Herein, we conducted a retrospective analysis to evaluate the safety and efficacy in ES-SCLC patients who received atezolizumab/durvalumab plus platinum–etoposide chemotherapy combined with thoracic RT in the first-line setting.

## Materials

### Patients

**Fig. 1:**
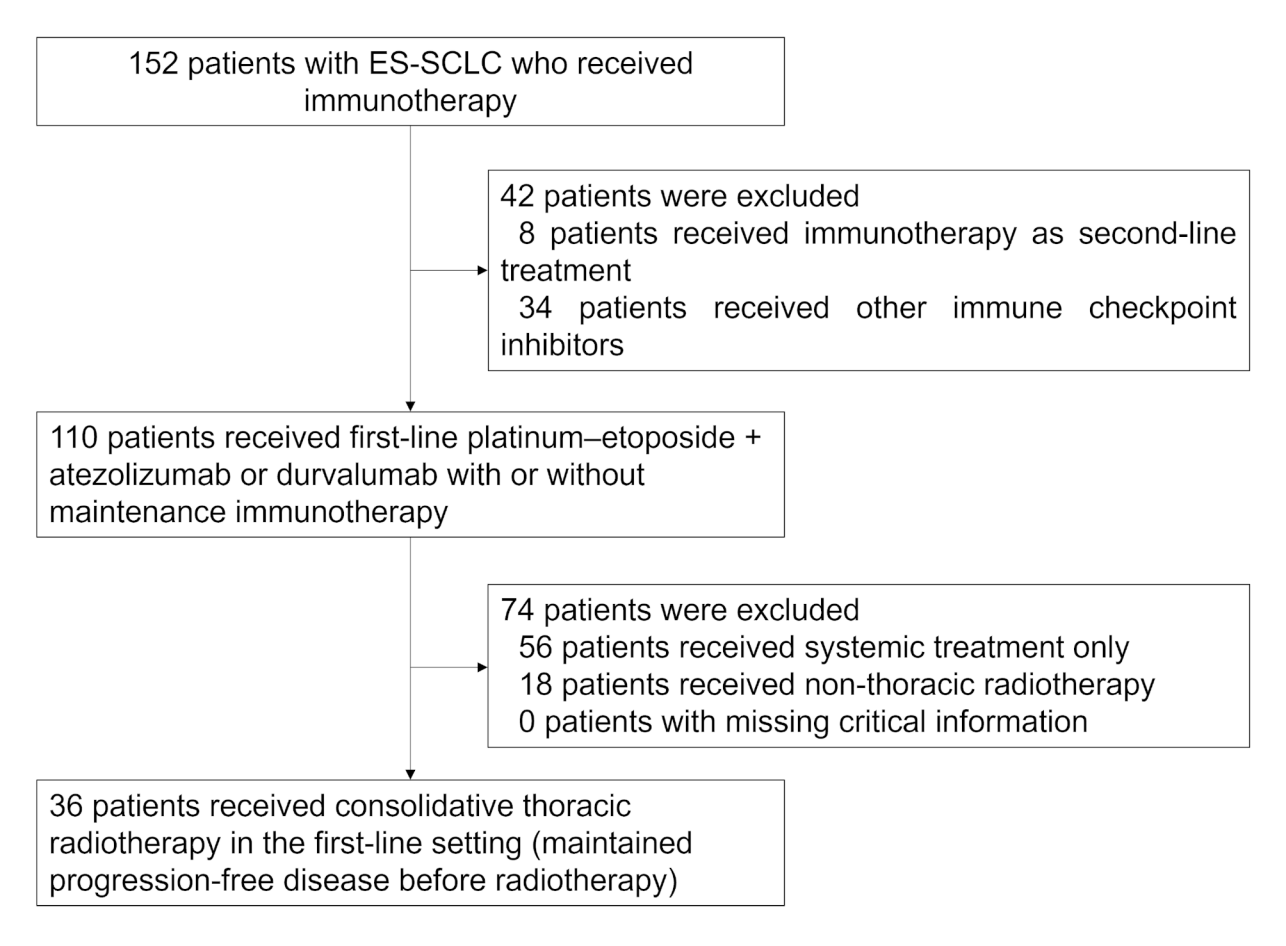
Diagram of patient’s selection process

This single-center retrospective study was conducted according to the Declaration of Helsinki and approved by Ethics Committee of Peking University Cancer Hospital (Approval number is not applicable) and identified from the hospital database all patients with SCLC who were treated in Peking University Cancer Hospital between 2019 and 2021. The inclusion criteria were as follows: (a) pathologically diagnosed with SCLC or mixed SCLC; (b) confirmed diagnosis of ES-SCLC according to the Veterans Administration Lung Study Group staging system [15] (a disease that is beyond the ipsilateral hemithorax and regional lymph nodes and could not be safely encompassed by a single radiation field); and (c) standard platinum–etoposide chemotherapy combined with atezolizumab/durvalumab immunotherapy in the first-line treatment and CTRT performed timely prior to tumor progression. The exclusion criterion was the presence of any autoimmune disorder or missing critical information (safety profile were regarded as critical). Finally, 36 patients were included in the analysis. Diagram of patient’s selection process was shown in Fig. 1. Data on baseline characteristics and details of treatment were fully collected.

### Treatment

First-line chemoimmunotherapy was administered as early as possible after diagnosis: four to six cycles of etoposide plus carboplatin or cisplatin combined with atezolizumab or durvalumab depending on patient performance status in the induction phase, followed by atezolizumab or durvalumab maintenance therapy until disease progression or unacceptable adverse event occurs. Thoracic radiation therapy was delivered in curative intent or symptom control. For patients who responded to chemoimmunotherapy but with persistent thoracic disease or bulk disease result in compression or dyspnea, thoracic RT is an appropriate treatment. Treatment plan, target volume definition, dose prescription and fraction, and RT technique were at the discretion of an experienced radiation oncologist. A four-dimensional physical planning was applied to optimized dose distribution and to protect organs at risk such as the lungs, esophagus, and heart. Precise RT was performed by professional radiation team.

### Outcomes

The primary outcome was safety of combination therapy, and the secondary outcomes included OS and progression-free survival (PFS). Adverse events were graded according to Common Terminology Criteria for Adverse Events v4.0. OS was defined as interval from initial chemotherapy to death from any cause. PFS was defined as duration from initial therapy to progression of any lesion or death from any cause, whichever happened first. Treatment effect was assessed by the Response Evaluation Criteria in Solid Tumors (RECIST) 1.1.

### Statistical analysis

All statistical analysis was performed with IBM Statistical Package for the Social Sciences v24. Descriptive statistics was used to characterize the patient characteristics. The Kaplan–Meier method was used to estimate OS and PFS distribution.

## Results

### Patient characteristics

We analyzed 36 ES-SCLC patients (median age, 63 years [range, 35–84 years], 86% male, 72% smokers) who received CTRT and showed no progress after chemoimmunotherapy between December 2019 and November 2021. Baseline characteristics are outlined in Table 1. Eastern Cooperative Oncology Group performance status for all patients was ≤ 2, with the majority scoring 0–1 (94%). Thirty-four patients had SCLC, while two patients had mixed SCLC (large cell neuroendocrine cancer mixture and mucinous adenocarcinoma). There were up to four metastatic sites at diagnosis, whereas most patients had multiple lesions at single metastatic site. Twenty-two (61%) patients had one metastatic site, while eight (22%) and six (17%) patients had two and three to four sites, respectively. Of all included patients, 58% presented with thoracic metastases including bilateral pulmonary and pleura metastases, 22% with liver metastasis, and 19% with brain metastasis; other locations included the adrenal gland and other intra-abdominal sites.

**Table 1:** Baseline Characteristics of All Patients

Characteristic	No. of Patients (%) or Median (Range)	
Gender		
Male	31	86%
Female	5	14%
Median age, years	63	35–84
ECOG PS		
0	14	39%
1	20	55%
2	2	6%
Smoker	26	72%
Pathology		
SCLC	34	94%
Mixed-SCLC	2	6%
Number of metastatic sites		
1	22	61%
2	8	22%
3–4	6	17%
Locations of metastases		
Brain	7	19%
Liver	8	22%
Thoracic	21	58%
Bone	11	30%
Other	10	28%
Previous treatment	4	11%
Chemotherapy	4	/
Radiation therapy	3	/
Immunotherapy	0	/

ECOG PS, Eastern Oncology Collaborative Group performance status; SCLC, small-cell lung cancer

### Treatment modality

Thirty-two patients presented with ES-SCLC at initial diagnosis, and the other four were pretreated for limited-stage SCLC with a treatment-free interval of more than 6 months. Except for one patient lost to follow-up after two cycles of chemotherapy, all patients completed chemotherapy for the standard 4–6 cycles. Until the last follow-up, the median number of cycles of immunotherapy was six (range, 1–17 cycles), and seven patients are still under treatment. More patients used atezolizumab (78%) than durvalumab (22%). After initial chemoimmunotherapy, the treatment response showed 3% CR (one patient), 64% PR, and 33% SD. All patients performed CTRT to the primary and subsequently involved regional nodes; 56% of these patients received maintenance immunotherapy. Throughout the whole course, seventeen patients discontinued immunotherapy due to disease progression, and nine due to adverse effects. The median interval between chemoimmunotherapy and CTRT was 4.6 weeks, and more than 90% patients started CTRT within 9 weeks. The majority of patients had conventional fractionation RT QD regimen (60 Gy in 28 fractions for 5.5 weeks). Five patients had stereotactic body radiotherapy (SBRT) with 37.5–50 Gy in 4–5 fractions. Nine patients received accelerated hypofractionation QD regimen (45 Gy in 15 fractions), and six received hyperfractionated twice daily (BID) (45 Gy in 30 fractions BID for 3 weeks). The biological effective dose (BED) ranged from 52 to 113 Gy. Nine patients had brain RT, two of which were prophylactic cranial irradiation (PCI).

### Safety

Adverse events during RT with or without maintenance immunotherapy were listed in detail in Table 2. Among the 36 patients who showed signs of toxicity, none experienced grade 4 or 5 toxicities. Most adverse events were tolerable and self-limiting, which were easy to handle and could be managed in the outpatient setting. Only two patients (6%) with grade 3 toxic effect developed thrombocytopenia. The most common toxicities were hematologic and gastrointestinal related, more specifically were anemia and radiation esophagitis. The bigger concern was respiratory toxicity. There were five patients developed adverse event of lung after CTRT, with three (8%) had radiation pneumonitis (one of grade 1, two of grade 2), one had immune-related pneumonitis (grade 2) and one had pulmonary fibrosis. We also noticed that four patients withdrew immunotherapy due to immune-related pneumonitis in the induction chemoimmunotherapy period but successfully completed CTRT in the later course. The incidence of immune-related pneumonitis at any occasion was 14%. Uncommon toxicity including pleural reactive effusion, elevated urinary protein, allergic reaction, and encephalitis were considered as immune-related.

**Table 2:** Adverse events during radiotherapy

	Total	G1	G2	G3
General		15(42%)		
Fatigue	14(39%)	13(36%)	1(3%)	0
Radiation dermatitis	4(11%)	3(8%)	1(3%)	0
Hematologic		25(69%)		
Leucopenia	15(42%)	9(25%)	6(17%)	0
Neutropenia	9(25%)	7(19%)	2(6%)	0
Anemia	18(50%)	12(33%)	6(17%)	0
Thrombocytopenia	8(22%)	3(8%)	3(8%)	2(6%)
Respiratory		9(25%)		
Radiation pneumonitis	3(8%)	1(3%)	2(6%)	0
Immune-related pneumonitis before RT	4(11%)	4(11%)	0	0
Immune-related pneumonitis after RT	1(3%)	0	1(3%)	0
Pulmonary fibrosis	1(3%)			
Gastrointestinal		25(69%)		
Radition esophagitis	18(50%)	15(42%)	3(8%)	0
Nausea	11(31%)	11(31%)	0	0
Constipation	2(6%)	2(6%)	0	0
Elevated transaminase	4(11%)	4(11%)	0	0
Others		5(14%)		
Pleural reactive pleural effusion	1(3%)	1(3%)	0	0
Elevated urinary protein	1(3%)	1(3%)	0	0
Allergic reaction before RT	2(6%)	2(6%)	0	0

RT: radiotherapy; G1: grade 1; G2: grade 2; G3: grade 3

### Survival analysis

**Fig. 2:**
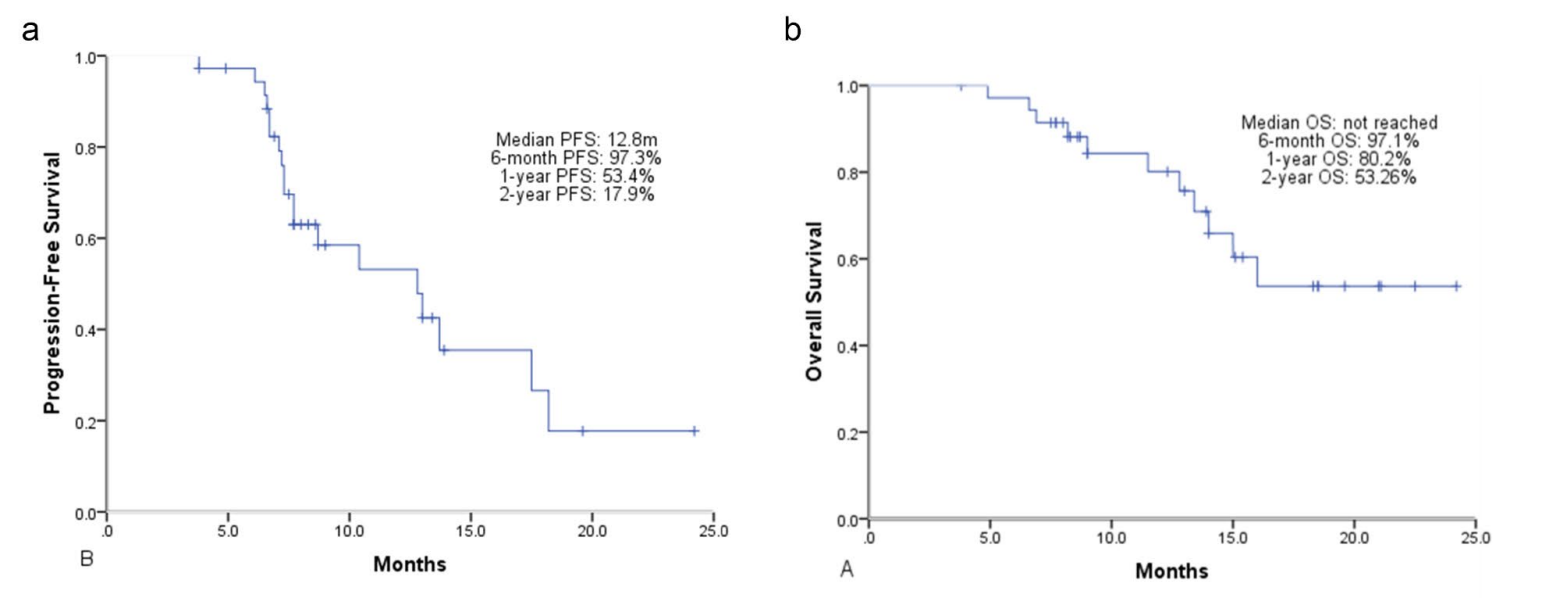
Kaplan–Meier curves showing (a) progression-free survival, (b). overall survival

At a median follow-up of 12.6 months (range, 3.8–24.8 months), 19 patients (53%) developed disease progression and 11 patients (31%) died from any cause. The median PFS was 12.8 months (95% confidence interval [CI], 7.5–18.1, Fig. 2a). The most common site of progression was brain and most of them were new metastatic lesions. Only one patient progressed in the irradiated field half year after radiation. The estimated PFS rate at 6-month, 1-year, and 2-year were 97.3%, 53.4% and 17.9%, respectively. Median OS was not reached (range, 3.9–24.8 months; Fig. 2b). The estimated OS rate at 6-month, 1-year, and 2-year were 97.1%, 80.2% and 53.3%, respectively. In the subgroup analysis, treatment response CR/PR group compared with SD showed near-significant longer PFS (13.7 vs. 7.7 months, p = 0.051) and OS (not reached vs. 13.4 months, p = 0.088). While BED > 60 Gy group compared with < 60 Gy show no discrepancy of PFS (13.0 vs. 10.4 months, p = 0.543) and OS (not reached vs. 15.0 months, p = 0.290).

## Discussion

In the era of immunotherapy, the data regarding safety and efficacy of immunotherapy combined with RT in ES-SCLC is scarce. In our retrospective study of ES-SCLC patients treated with chemo-atezolizumab/durvalumab followed by CTRT with maintenance immunotherapy or not in the first-line setting, we found a manageable safety profile and appreciable survival benefit with the addition of radiation. As RT-immunotherapy combination has been a part of new standard of care in many malignances, such as non-small cell lung cancer [12], the result of this analysis supports the further study on how to integrate thoracic RT into the chemoimmunotherapy backbone for ES-SCLC, the challenging disease with limited efficacy of existing therapeutic options and poor prognosis.

For ES-SCLC, thoracic RT was assessed in several randomized trials in patients who response to initial platinum-doublet chemotherapy. Jeremic et al. [6] firstly concluded that introduction of thoracic RT (54 Gy in 36 fractions over 18 days) in the cisplatin-etoposide chemotherapy offers promising results with improved survival compared with chemotherapy alone (17 vs. 11 months of median OS, p = 0.041). More recently, in the large CREST EORTC phase III study, 498 ES-SCLC patients without pleural or brain metastases who responded to four to six cycles of standard chemotherapy were randomized into thoracic RT (30 Gy in 10 fractions) + PCI and PCI alone [7]. The primary endpoint of 1-year OS for the thoracic RT group was higher than the control group but was not statistically different (33% vs. 28%, p = 0.066), while 2-year OS was found to be significantly different between the groups in a secondary analysis (13% vs. 3%, p = 0.004). Thoracic RT for ES-SCLC significantly improved 6-months PFS (24% vs. 7%, p = 0.001) and intrathoracic disease control (p < 0.0001) with very well tolerated toxic effects. And subgroup analysis indicated that patients with residual intrathoracic disease and less than three distant metastases benefit more from RT [16, 17]. Later, phase II trial RTOG 0937 by the Radiation Therapy Oncology Group intended to compare PCI alone to PCI + consolidative radiation to thorax and metastatic sites (45 Gy in 15 fractions). Unfortunately, the trial closed early due to the futility boundary for OS was crossed, but it did show that consolidative radiation obtained longer time to progression [8]. Regarding to the conflict data, CTRT for ES-SCLC should be considered carefully for the benefit population, and immunotherapy-based comprehensive therapy modality needs further evaluation [18].

IMpower133 was the first major advance that demonstrated significant longer OS (12.3 vs. 10.3 months of median OS; 51.7% vs. 38.2% of 1-year OS, p = 0.007) and PFS (5.2 vs. 4.3 months of median PFS; 12.6% vs. 5.4% of 1-year PFS, p = 0.02) of combining atezolizumab (humanized monoclonal PD-L1 inhibitor) and chemotherapy than chemotherapy alone with placebo in ES-SCLC [4]. Immune-related pneumonitis was 2% of all grades and 0.5% of grade 3–4. Similar results were obtained in CASPIAN trial [5], the addition of durvalumab (humanized monoclonal PD-L1 inhibitor) significantly improved OS (13 vs. 10.3 months of median OS; 54% vs. 40% of 1-year OS, p = 0.0047) compared with platinum–etoposide alone.

As described above, although several randomized trials have shown benefits to thoracic RT or immunotherapy addition to the chemotherapy, combination with chemoimmunotherapy and thoracic RT is a prudent choice with concerns of understudied safety profiles. Immunotherapy carries specific toxicity risks and may incur severe adverse events in combination with RT, especially with regard to pulmonary toxicity such as pneumonitis and respiratory failure, which might be life-threatening [19]. Hence, clarifying its safety is essential for further therapeutic decision, which is also the primary intent of our study. An analysis of 3 single-institutional phase I/II trials demonstrated that the combined regimen is safe for lung cancer patients in the short term independent of techniques and dosimetry of thoracic RT [20]. For ES-SCLC, Welsh et al. [21] conducted a single-arm Phase I trial (NCT02402920) assessing the safety of pembrolizumab and thoracic RT in concurrent schedule in 33 patients who completed six induction cycles of chemotherapy. With prescription dose as 45 Gy in 15 daily fractions and dose-escalation of pembrolizumab, there were no grade 4–5 adverse events and those possibly related to protocol therapy were limited to grade 1–2, most commonly esophagitis (26%), fatigue (24%), dysphagia (21%) and no pneumonitis. Another phase I/II trials (NCT03043599) indicated that maintenance ipilimumab and nivolumab after CTRT (30 Gy in 10 fractions) presented adverse events consistent with the known immune-related toxicity profile [22]. Besides, a multi-institutional case series of 20 patients also showed that first-line chemoimmunotherapy with atezolizumab followed by CTRT is safe with only 5% grade 2 esophagitis [23]. In our study, atezolizumab or durvalumab combined with CTRT in the first-line setting carried very low risk of severe toxicity likewise and similar safety profile. Grade 1–2 toxicity were mainly esophagitis (50%), marrow suppression and fatigue (39%). Radiation pneumonitis were 8% and grade 3 toxicity occurred in merely two patients (6%) with thrombocytopenia, who recovered soon after symptomatic treatment. The overall adverse effects rate is higher than above studies probably due to the higher CTRT dose for curative intent. What’s more, the incidence of immune-related pneumonitis (14%) in the real word is higher than that reported in literature (2%) as well [4], but without negative effects to the later CTRT. Despite of different ICI and RT delivery, it’s possible to believe that immunotherapy combined radiation does not overtly increase sever toxicity incorporating aforesaid studies.

Regarding clinical outcomes, at a median follow-up of 12.6 months, the median PFS was 12.8 months and OS was not reached, which is impressive and exceeded historic expectations [4, 5]. What we have to admit is that our study is small sample and single-arm, making it difficult to compare directly. And there were some heterogeneities of the study population, such as different cycles of induction chemoimmunotherapy, RT techniques, doses and immune-RT interval due to the retrospective nature, which blended the result. However, it’s meaningful to characterize factors contributing to better survival. Han et al. [24] identified different outcomes by radiation time and dose in ES-SCLC patients who received thoracic RT combined with chemotherapy. The result showed that RT combined with chemotherapy significantly improved OS, PFS and local recurrence-free survival before and after matching. Moreover, early RT especially within 6 cycles of chemotherapy prolong local recurrence-free survival (p = 0.001) and hyper-fractioned scheme (45 Gy in 30 fractions twice per day) has survival advantage over 60 Gy/30 fractions daily, which in line with Luan’s finding [25]. However, Stanic et al. concluded that higher dose resulted in better OS [26]. In our study, subgroup analysis showed no discrepancy of PFS and OS between BED > 60 Gy and < 60 Gy. That means higher doses may be not necessary for survival, which is in consistent with Han’s finding to some extent [24]. However, further study is warrant due to the small subgroup analysis. What’s more, we also found that patients with good response to induced chemoimmunotherapy had a trend of longer PFS, which was helpful to identify who would benefit most from thoracic RT. Further, we are interested in the application of SBRT in such population with the advantage of short treatment time, avoidance of immune maintenance disruption, and enhanced synergistic effect. Meanwhile, several relevant trails are ongoing and safety as well as efficacy results are expected in years to come. NCT03923270 [27] and ACTRN12621000586819 [28] focus on durvalumab combined with 30 Gy in 10 fractions daily thoracic RT, and phase III MAURIS trail (NCT04028050) [29] will evaluate atezolizumab in combination with carboplatin/etoposide and thoracic RT is allowed. The randomized RAPTOR trial (NCT04402788) [13] and TREASURE study (NCT04462276) [14] will evaluate thoracic RT with atezolizumab for ES-SCLC. These trails will provide more data on how best to utilize RT based on the standards of care in the chemoimmunotherapy era.

The present study had several limitations aside from the aforementioned problem. First, retrospective design coupled with small sample size in single center cause inevitable patient selection biases and limited the subgroups analysis. Second, patient baseline characteristics were somewhat heterogeneous. Four patients diagnosed as limited-stage SCLC initially but all the treatment-free interval exceeded 6 months. Third, the treatments were not uniform and may affect the interpretation of the result, including different radiation timing, and dose/fractions. In light of these defects, our findings should be tested in further studies; nevertheless, we would like to underline that the safety and efficacy support further investigation.

## Conclusions

In this retrospective analysis of 36 patients with ES-SCLC received standard platinum–etoposide chemotherapy combined with atezolizumab/durvalumab immunotherapy followed by CTRT, we found a manageable safety profile and appreciable survival benefit, which are comparable to the published trials. Further studies with prospective design are warranted.

## Data Availability

The datasets used and/or analysed during the current study are available from the corresponding author on reasonable request.

## List of abbreviations

BED: biological effective dose
CTRT: consolidative thoracic radiotherapy
ES-SCLC: extensive-stage small-cell lung cancer
ICI: immune checkpoint inhibitors
OS: overall survival
PCI: prophylactic cranial irradiation
PFS: progression-free survival
RECIST: Response Evaluation Criteria in Solid Tumors
RT: radiotherapy
SBRT: stereotactic body radiotherapy
SCLC: small-cell lung cancer
